# Linoleic acid concentration at the air-liquid interface is key for optimal incorporation into skin model lipidome

**DOI:** 10.3389/fphys.2026.1838020

**Published:** 2026-06-22

**Authors:** Andréa Tremblay, Angélina Larouche, Mélissa Simard, Élizabeth Dumais, Pierre Julien, Nicolas Flamand, Roxane Pouliot

**Affiliations:** 1 Faculté de pharmacie, Université Laval, Québec, QC, Canada; 2 Axe Médecine Régénératrice, Centre de Recherche du CHU de Québec-Université Laval, Québec, QC, Canada; 3 Centre de Recherche en Organogénèse Expérimentale de l’Université Laval/LOEX, Québec, QC, Canada; 4 Centre de recherche de l’Institut universitaire de cardiologie et de pneumologie de Québec, Département de médecine, Faculté de médecine, Université Laval, Québec, QC, Canada; 5 Axe d’Endocrinologie et de Néphrologie, Centre de Recherche du CHU de Québec–Université Laval, Québec, QC, Canada

**Keywords:** essential fatty acids, lipid metabolism, lipidomics, skin model, tissue engineering, linoleic acid

## Abstract

Linoleic acid is an essential fatty acid required for skin barrier formation. However, linoleic acid-deficient media remains widely used in cell culture and reconstructed skin production, potentially contributing to suboptimal barrier function in skin models. In this study, we investigated the effects of varying linoleic acid concentrations in culture media on lipid composition, bioactive lipid mediator production, and gene expression of lipid-metabolizing enzymes in self-assembled skin substitutes. High-concentration linoleic acid significantly improved barrier function, as evidenced by reduced testosterone permeability, and enhanced incorporation into phospholipids, triacylglycerols, and diacylglycerols. Unexpectedly, ω-hydroxy-sphingosine ceramide proportions were reduced, reflective of downregulation of key enzymes in ω-O-acylceramide synthesis. These findings reveal a complex interplay between linoleic acid availability, lipid remodeling, and enzymatic regulation, offering new insights into optimizing reconstructed skin models for improved barrier function and lipid homeostasis.

## 1 Introduction

The skin barrier has garnered increasing attention, particularly following the emergence of the Epithelial Barrier Theory, which links the rise in chronic inflammatory diseases to compromised epithelial integrity driven by urbanization and modern lifestyles (Sun et al., 2024). In this context, the stratum corneum plays a central role, acting as the primary permeability barrier of the skin. Its function relies on a highly organized extracellular lipid matrix, composed mainly of ceramides, cholesterol and free fatty acids, arranged in lamellar bilayers that limit transepidermal water loss and the penetration of exogenous compounds (Vietri Rudan and Watt, 2021). The integrity and organization of this lipid matrix are therefore essential for maintaining skin homeostasis, and even subtle alterations in lipid composition can lead to impaired barrier function (Skolová et al., 2013; Uche et al., 2019b; Uche et al., 2021).

Despite advances in tissue engineering, reconstructed human skin models often exhibit higher permeability than native human skin (NHS), limiting their utility in toxicity testing and drug absorption studies (Asbill et al., 2000; Netzlaff et al., 2005; Netzlaff et al., 2007). This limitation has been largely attributed to differences in lipid composition and organization within the stratum corneum, particularly a reduced content of key ceramide subclasses and altered lipid lamellar structures (Tfayli et al., 2014). As a result, improving the lipid profile of reconstructed skin remains a major challenge for the development of physiologically relevant *in vitro* models. Three-dimensional human skin substitutes provide a controlled platform to dissect the molecular and lipidomic effects of specific culture conditions, such as fatty acid supplementation, on epidermal development and function.

This study provides a comprehensive lipidomic analysis of a 3D skin model, evaluating the impact of linoleic acid (LA) supplementation on ceramide classes, triacylglycerol (TAG) and phospholipid (PL) composition, as well as monoacylglycerols (MAGs), diacylglycerols (DAGs) and other lipid mediators. While several groups have modified the lipid composition of culture media, notably by supplementing with palmitic acid or a fatty acid mix, our previous work focused on addressing essential fatty acid deficiency to improve cellular lipid homeostasis (Thakoersing et al., 2015; Mieremet et al., 2019; Simard et al., 2019; Simard et al., 2021). This is because LA is recognized to support skin barrier formation through its incorporation into ω-O-acylceramides, which are essential for the formation of the cornified lipid envelope (CLE), a scaffold critical for lipid bilayer organization in the stratum corneum (Opálka et al., 2022).

We hypothesized that increasing LA availability during the production of skin substitutes would enhance the formation of ω-O-acylceramides and improve the overall organization of the epidermal lipid matrix, leading to a lipid profile more representative of NHS and, consequently, to improved barrier function. By clarifying the role of both low and high concentrations of LA in shaping the epidermal lipidome, this study aims to contribute to the optimization of skin models for biomedical and industrial applications, leveraging a well-established and thoroughly characterized skin model (Michel et al., 1995; Pouliot et al., 1999; Lavoie et al., 2013; Leroy et al., 2014; Larose et al., 2020).

## 2 Materials and methods

### 2.1 Ethics

This study was approved by the institutional review board of Université Laval and conducted in accordance with the Declaration of Helsinki and the guidelines of the Research Ethics Committee of the CHU de Québec-Université Laval.

### 2.2 Cell culture

The tissue-engineered skin substitutes were produced with healthy fibroblasts and keratinocytes extracted from breast reduction skin biopsies of three Caucasian women aged 46, 47 and 49 years old, using a method based on thermolysin, trypsin and collagenase digestion, as previously described elsewhere (Germain et al., 1993). All experimental results were obtained by analyzing the skin substitutes produced from these three donors.

### 2.3 Tissue-engineered skin substitute reconstruction

Skin substitutes were produced according to the self-assembly method described elsewhere (Simard et al., 2019). Primary human fibroblasts (passage 6) were cultured for 26 days in 6-well plates (1,25 × 10^4^ cells/cm^2^) with Dulbecco’s modified Eagle’s medium (DME) (Gibco, Life Technologies, New York, NY, US) supplemented with 10% Fetal Bovine Serum (Seradigm, Radnor, PA, USA), 50 μg/ml ascorbic acid (Sigma, Oakville, ON, CAN), 60 μg/ml penicillin G (Sigma, Oakville, ON, CAN) and 25 μg/ml gentamicin (Schering, Pointe-Claire, QC, Canada). Two of the resulting fibroblast sheets were superimposed and cultured for 2 days in a 100 mm Petri dish to form a dermal equivalent. Matching primary human keratinocytes (passage 3) were then seeded at 1,2 × 10^6^ cells per dermal equivalent. The newly seeded skin substitutes were cultured in submerged conditions for one week in DME mixed with Ham’s F12 medium (3:1) (DMEH) (Gibco, Life Technologies, New York, NY, US) supplemented with 5% FetalClone II serum (Hyclone, Logan, UT, US), 5 μg/ml insulin (Sigma, Oakville, ON, CAN), 0.4 μg/ml hydrocortisone (Galenova, St-Hyacinthe, QC, CAN), 0.4 μg/ml isoproterenol (Sigma, Oakville, ON, CAN), 10 ng/ml human epidermal growth factor (EGF) (Ango Inc., San Ramon, CA, US), 60 μg/ml penicillin and 25 μg/ml gentamicin. The skin substitutes were raised to the air-liquid interface and cultured for 21 additional days without EGF (Michel et al., 1999). The cell culture media were changed three times a week and the skin substitutes were kept at 37°C in an 8% CO_2_ atmosphere (L'Heureux, 1998).

### 2.4 Fatty acid supplementation of cell culture media

**Figure 1:**
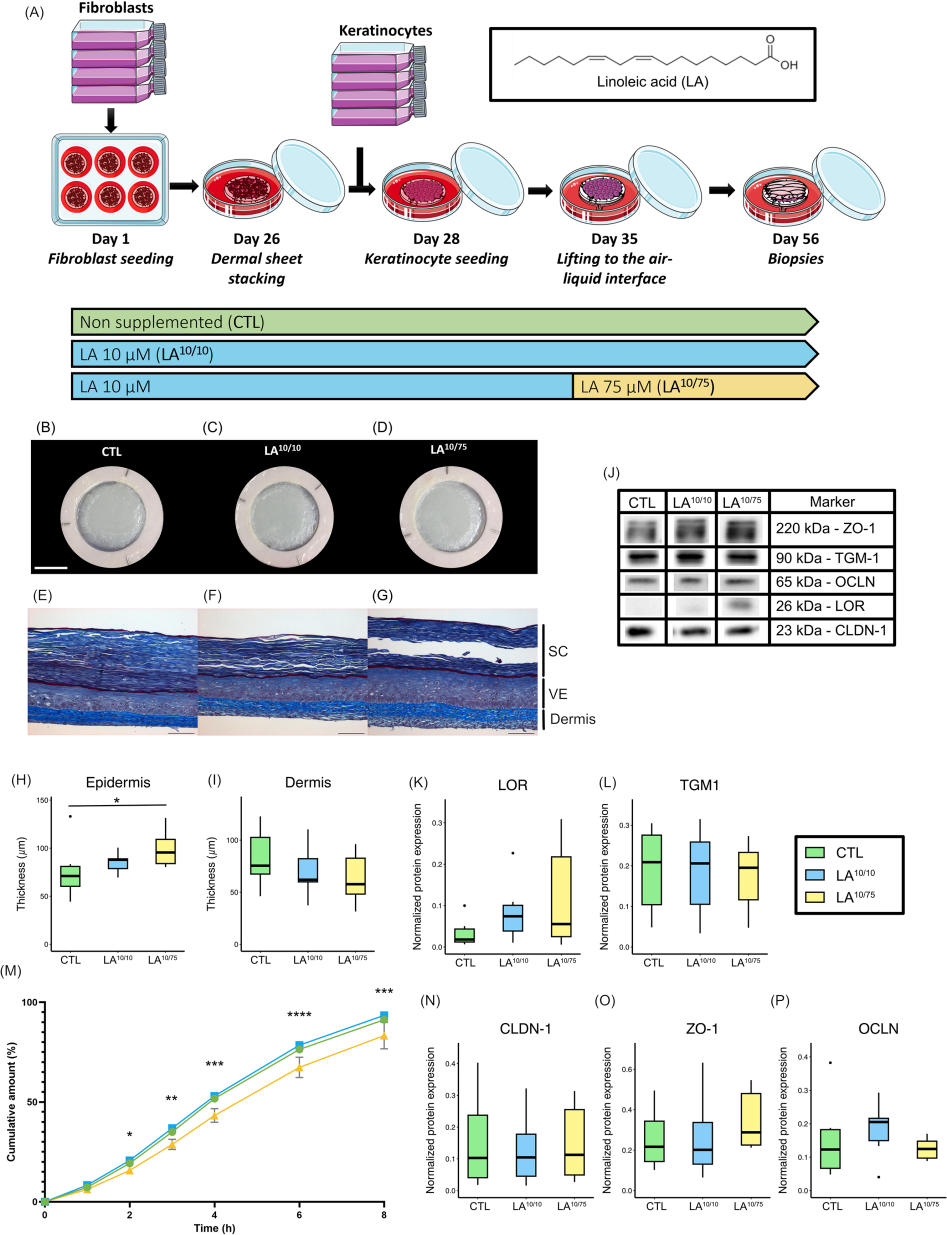
Structural and functional characterization of skin substitutes following linoleic acid supplementation. **(A)** Schematic representation of the self-assembly method and culture conditions used to generate skin substitutes with linoleic acid structure. **(B–D)** Macroscopic appearance of the skin substitutes. **(E–G)** Histological analysis of 5 μm-thick cross-sections stained with Masson’s trichrome. Quantification of **(H)** epidermal and **(I)** dermal thickness from Masson’s trichrome-stained sections. **(J)** Western blot analysis of protein expression for **(K)** loricrin (late differentiation marker) and **(L)** transglutaminase-1 (early differentiation marker), normalized to total protein content. **(M)** Cumulative testosterone permeation through the skin substitutes over 8 hours using Franz diffusion cells. Data are presented as mean ± standard error of the mean (SEM). *p*-values were derived from Two-way ANOVA followed by Tukey’s *post-hoc* test (N = 3 donors, n=5 skin substitutes per donor). Semi-quantitative western blot analysis of tight junction proteins: **(N)** claudin-1, **(O)** zonula occludens-1, and **(P)** occludin in the epidermal compartment normalized to total protein content, uncropped membranes can be found in supplementary **Supplementary Figure 5**. **(H–J, K, L, N–P)** *p*-values were derived from a linear mixed-effects model followed by Tukey’s *post hoc* test (N = 3 donors, n=2 skin substitutes per donor). Significance levels: *p-value < 0.05; **p-value < 0.01; ***p-value < 0.001; ****p-value < 0.0001. Scale bars: **(B–D)** 1 cm; **(E–G)** 100 μm.

Individual stock solutions were prepared by dissolving LA (Sigma, Oakville, ON, CAN) in 99% ethanol (EtOH) (Greenfield Global, Brampton, ON, CAN). During media preparation, calculated volumes of the LA stock solution were first evaporated and then incorporated into serum containing naturally abundant bovine serum albumin to enhance fatty acid solubility, yielding final LA concentrations of either 10 μM or 75 μM depending on the supplementation (Alsabeeh et al., 2018). Skin substitutes were produced under three different conditions, including the unsupplemented controls (CTL). Substitutes LA^10/10^ were supplemented with 10 μM LA all throughout the production of the reconstructed skin, as published in 2019 (Simard et al., 2019). Substitutes LA^10/75^ were initially supplemented with 10 μM LA until day 35, at which point they were raised to the air-liquid interface, and the LA concentration was increased to 75 μM for the remainder of the culture period. A schematic representation of the different fatty acid supplementations of cell culture media can also be found in **Figure 1**. The culture media were changed three times a week.

### 2.5 Percutaneous absorption

The permeability of *in vitro* skin substitutes to testosterone was assessed using static Franz diffusion cells (5 mL volume, 0.63 cm^2^ surface area; Crown Glass, Somerville, NJ, USA), in accordance with the Organization for Economic Cooperation Development (OECD) guidelines (OECD, 2004). Clamps were holding the donor and receiver compartments of the Franz cells in which the skin substitutes were placed in between. The receptor compartments were filled with a 0.1 M phosphate buffered saline (PBS) solution at pH 7.4 and a stirring bar was added to ensure homogeneity. The Franz diffusion cells were placed on a heating bench (LOGAN Instruments Corp., Somerset, NJ, USA) to maintain the temperature of the receptor compartments at 37°C, keeping the skin samples at approximately 32°C. The initial time point (0) started when 100 μl of a 4 mg/mL testosterone solution in EtOH/water (1:1 v/v) was added to each donor compartment. ParaFilm was applied over the donor compartments to ensure occlusion. Samples were collected from the receptor compartments using a 5 mL syringe lengthened with a catheter (3 ½ Tom Cat Length 4 ½) at various time points (1 h, 2 h, 3 h, 4 h, 6 h, 8 h and 24 h). The samples were then filtered through a 0.22 μm filter and stored at –4°C until analysis.

Testosterone samples were assayed by an in-house-developed UPLC-UV method at 248 nm using a Waters Acquity UPLC system with a Water photodiode array (PDA) detector and a thermostatted autoinjector (Acquity UPLC H-Class System, Waters, Mississauga, ON, Canada). Separation of testosterone from the samples was achieved using a BEH C18, Waters column (50 mm × 2.1 mm, 5 μm, Mississauga, ON, Canada) maintained at 50°C. The mobile phase consisted of a gradient concentration of acetonitrile in water with 0.1% trifluoroacetic acid (TFA) eluted at a flow rate of 0.6 mL/min. Under these conditions, testosterone was eluted at 0.81 min. Data collection and peak integration were carried out using Empower 2 software (Waters, Mississauga, ON, Canada).

### 2.6 Histological analysis

Skin substitutes were fixed using 3,7% formalin (VWR, Montreal, QC, Canada) and subsequently embedded in paraffin. 5 μm-thick sections were cut and stained with Masson’s Trichrome. For each donor, two substitutes were analyzed (n=2). The stained skin sections were observed using a Zeiss microscope equipped with an AxioCam ICc1 (Carl Zeiss Meditec, AG, Oberkochen, Germany). The thickness of the epidermis was measured using ImageJ software (National Institutes of Health (NIH), Bethesda, USA, https://imagej.net/ij) by collecting ten measurements from three different sections of each stained biopsy.

### 2.7 Western blot analysis

The epidermis was separated from the dermis using a scalpel and forceps, quick-frozen, then stored at -80°C. Samples were crushed with a Cryomill MM400 (Retsch^®^) before protein extraction with 500 μL of RIPA buffer containing the protease inhibitor cocktail cOmplete (Roche, Mannheim, Germany). The samples were incubated on ice for 20 min, followed by centrifugation at 12,000 × g for 20 minutes at 4°C. The extracts were quantified using Pierce BCA Protein Assay Kit (Thermo Fisher Scientific).

The expression levels of CLDN-1, ZO-1, OCLN, LOR and TGM-1 relative to the total proteins of each sample were compared between all conditions using Western blot analysis. See **Supplementary Table 2** for primary antibodies information. A total of 20 μg of proteins were loaded onto 12% reducing SDS-PAGE gels. After electrophoresis, gels were transferred onto Immun-Blot PVDF membranes (Bio-Rad Laboratories, Mississauga, ON, Canada) at 20 Volts and 4°C overnight. The membranes were blocked with Tris-buggered saline 0.1% Tween-10 and 5% non-fat powdered milk (BioBasic Inc., Markham, ON, Canada) for 1 h, followed by incubation with primary antibodies (see **Supplementary Table 2** for incubation time of each primary antibody), and then with secondary antibodies for an additional hour. Depending on the source of primary antibodies, either anti-mouse HRP (1:60,000, 115-035-003, Jackson Immuno Research Laboratories Inc., West Grove, PA, USA) or anti-rabbit HRP (1:60,000, 111-035-003, Jackson Immuno Research Laboratories Inc.) secondary antibodies were used.

### 2.8 Real-time reverse transcription quantitative PCR

Separated epidermal samples from skin substitutes were lyzed with a 0.5 mg/mL proteinase K (BioBasic Inc.) solution in 10 mM Tris-HCl, 10 mM EDTA, 2% SDS (pH 9.0), and homogenized using a mortar. DNA was eliminated using QIAzol (Qiagen, Toronto, ON, Canada) and RNA was precipitated with ethanol before RNA isolation with RNeasy Mini Kit (Qiagen). RNA concentrations were determined using a NanoDrop 2000c Spectrophotometer (Thermo Fisher Scientific). Reverse transcription was completed using the Quantitect reverse transcription kit (Qiagen) before diluting the samples to the desired concentration with DNase- and RNase-free water (Thermp Fisher Scientific). The qPCR reaction was done with SYBRGreen 5X Master Mix (Thermo Fisher Scientific) and 12,5 ng of cDNA per reaction. The qPCR reaction and analysis were performed using a LightCycler^®^ 480 II (Roche). Amplification was performed using the following thermal cycling protocol: uracil-DNA glycosylase (UDG) activation at 50 °C for 2 min, followed by initial denaturation at 95°C for 2 min. The reaction then proceeded through 40 cycles of denaturation at 95°C for 10 s, annealing at 54–59°C (see **Supplementary Table 3**) for 10 s, and extension at 72°C for 15 s. All reactions were conducted in duplicate to ensure reproducibility. GAPDH and ACTINβ were used as reference gene controls and mRNA expression was normalized to GAPDH expression. The primers for all the genes analyzed were ordered from IDT (Coralville, IA, USA) and sequences are listed in **Supplementary Table 3**.

### 2.9 Lipid extraction for mass spectrometry lipidomics

Mass spectrometry-based lipid analysis was performed by Lipotype GmbH (Dresden, Germany) as described elsewhere (Surma et al., 2021). Skin samples were homogenized in PBS to reach a final concentration of 5 mg/mL and stored at -80°C before lipid extraction using a chloroform/methanol method (Ejsing et al., 2009). Samples were spiked with an internal lipid standard mixture containing ω-hydroxy-sphingosine 18:1;2/32:0;0/18:2;0 (EOS D9), nonhydroxy-sphingosine 18:1;2/18:0;0 (NS D3), cholesterol ester 16:0 D7 (CE), diacylglycerol D5 17:0/17:0 (DAG) and triacylglycerol D5 17:0/17:1/17:0 (TAG). Following the extraction, the organic phase was transferred to an infusion plate and dried in a speed vacuum concentrator. The dried extracts were then re-suspended in an acquisition mixture of 7.5 mM ammonium acetate in chloroform/methanol/propanol (1:2:4 v/v/v). All liquid handling steps were performed using Hamilton Robotics STARlet robotic platform with the Anti Droplet Control feature for organic solvents pipetting.

### 2.10 MS data acquisition

Samples were analyzed by direct infusion using a QExactive mass spectrometer (Thermo Fisher Scientific, Rockford, IL, USA) equipped with a TriVersa NanoMate ion source (Advion Biosciences). Data acquisition was performed in both positive and negative ion modes, with a resolution of R_m/z=200_ = 280000 for MS and R_m/z=200_ = 17500 for MS/MS, all in a single acquisition. MS/MS fragmentation was initiated by an inclusion list that covered the corresponding MS mass ranges scanned in 1 Da increments (Surma et al., 2015). MS and MS/MS data were combined to monitor CE, DAG and TAG ions as ammonium adducts, along with all ceramide classes as acetate adducts.

### 2.11 Data analysis and post-processing

Data were analyzed by Lipotype with in-house developed lipid identification software based on LipidXplorer (Herzog et al., 2011; Herzog et al., 2012). Data post-processing and normalization were conducted using an in-house developed data management system. Only lipid identifications with a signal-to-noise ratio greater than 5 and a signal intensity 5-fold higher than in corresponding blank samples were considered for further data analysis.

### 2.12 Gas chromatography (GC-MS)

Gas chromatography was performed to analyze the fatty acid contents of the PL and TAG fractions of the epidermis. The epidermis of skin substitutes was first separated from the dermis using forceps and scalpels. Epidermal lipids were then extracted following a modified Folch method and using a chloroform/methanol mixture (2:1 v/v) (Julien et al., 2006). Lipid classes were separated by thin layer chromatography using an isopropyl ether/acetic acid mixture (96:4 v/v) until the solvent front reached the middle of the plate. The fatty acids of the isolated PLs and TAGs were subsequently methylated. Fatty acid profiles were obtained through gas chromatography, using a HP5890 gas chromatograph (Hewlett-Packard, Toronto, ON, Canada) composed of an HP-88 capillary column (100 mm × 0.25 mm internal diameter × 0.20 μm film thickness; Agilent Technologies, Santa Clara, CA, USA) coupled with a flame ionization detector, as described elsewhere (Gevariya et al., 2019).

### 2.13 Liquid chromatography-tandem mass spectrometry (LC-MS/MS)

The epidermis was mechanically separated from the dermis with forceps and scalpels. Each sample was then pulverized into a fine powder using a Cryomill MM400 (Retsch^®^, Newtown, PA, US). The powder obtained was suspended in 500 μL Tris-HCL 50 mM (pH 7) and immediately denatured in one volume of methanol at -20°C containing internal standards. Epidermal lipids were extracted using an acidified methanol chloroform technique, as described previously (Manca et al., 2020). Samples were reconstituted in 60 μL of a 50/50 mixture of LC solvent A (H_2_O containing 0.05% acetic acid and 1 mM NH_4_^+^) and solvent B (acetonitrile/H_2_O, 95/5, v/v, with 0.05% acetic acid and 1 mM NH_4_^+^). 40 μL of solvent B was injected onto a RP/HPLC column (Kinetex C8, 150 x 2.1 mm, 2,6 μm, Phenomenex), and lipids were separated using the LC program described by Everard et al. (2019). Quantification was performed by generating calibration curves using pure standards and analyzing on the LC-MS/MS system three times. The slope was then calculated using the ratio between the peak areas of the compound and its corresponding standard. Due to acyl migration from the *sn*-2- to the *sn*-1 (Uche et al., 2019b) position naturally occurring in MAGs, the data is presented as the combination of MAG isomers (1/2-MAG).

### 2.14 Statistical analysis

Data are presented as median ± min/max values within 1.5 × interquartile range (IQR) from the first and third quartile, except when stated otherwise. Statistical analyses of fatty acid lipid profiles and bioactive lipid mediators were conducted using a mixed model effect followed by Tukey’s *post-hoc* test if data distributions passed the Shapiro-Wilk normality test, or a Friedman test if they did not. To account for potential outliers, a robust linear mixed-effects model was employed when appropriate. P-values < 0.05 were considered statistically significant and values approaching significance are reported explicitly. All statistics calculations were performed using RStudio (Posit Software, PBC, Boston, MA, USA) and graphs were produced with either RStudio or Prism version 10 software (Graphpad Sowftware, La Jolla, CA, USA) and LipotypeZoom (Lipotype GmbH).

## 3 Results

The establishment of our reconstructed skin model lasts 56 days, the model being immersed during the first 35 days, then placed into an air-liquid interface (ALI) from days 35-56. Moreover, we previously showed that supplementing fatty acids at a concentration greater than 20 μM during the immersed phase altered the integrity of the epidermis, a phenomenon that is not observed during the ALI phase (Simard et al., 2019). For these reasons, we established a supplementation involving a LA supplementation at 10 μM during the immersed phase as well as an LA supplementation at a higher concentration during the ALI phase (75 μM; LA^10/75^) (**Figure 1A**). These were compared with non-supplemented controls (CTL) and continuous supplementation at 10 μM (LA^10/10^).

### 3.1 Epidermal morphology and differentiation across culture conditions

The three experimental conditions yielded a uniformly stratified epidermis (**Figures 1E–G**). The epidermal structure was consistent, with a well-defined stratum corneum and comparable morphological features across all culture conditions (**Figures 1B–D**). The thickness of the viable epidermis and the dermis were measured, and a significant increase in the thickness of the living epidermis was noted for the LA^10/75^ group compared with other conditions (**Figures 1H, I**). Additionally, immunoblot of loricrin (LOR) and transglutaminase-1 (TGM-1) indicate that these differentiation markers are comparable in all experimental conditions (**Figures 1J–L**).

### 3.2 *In vitro* barrier against testosterone is improved with high LA concentrations

**Figure 2:**
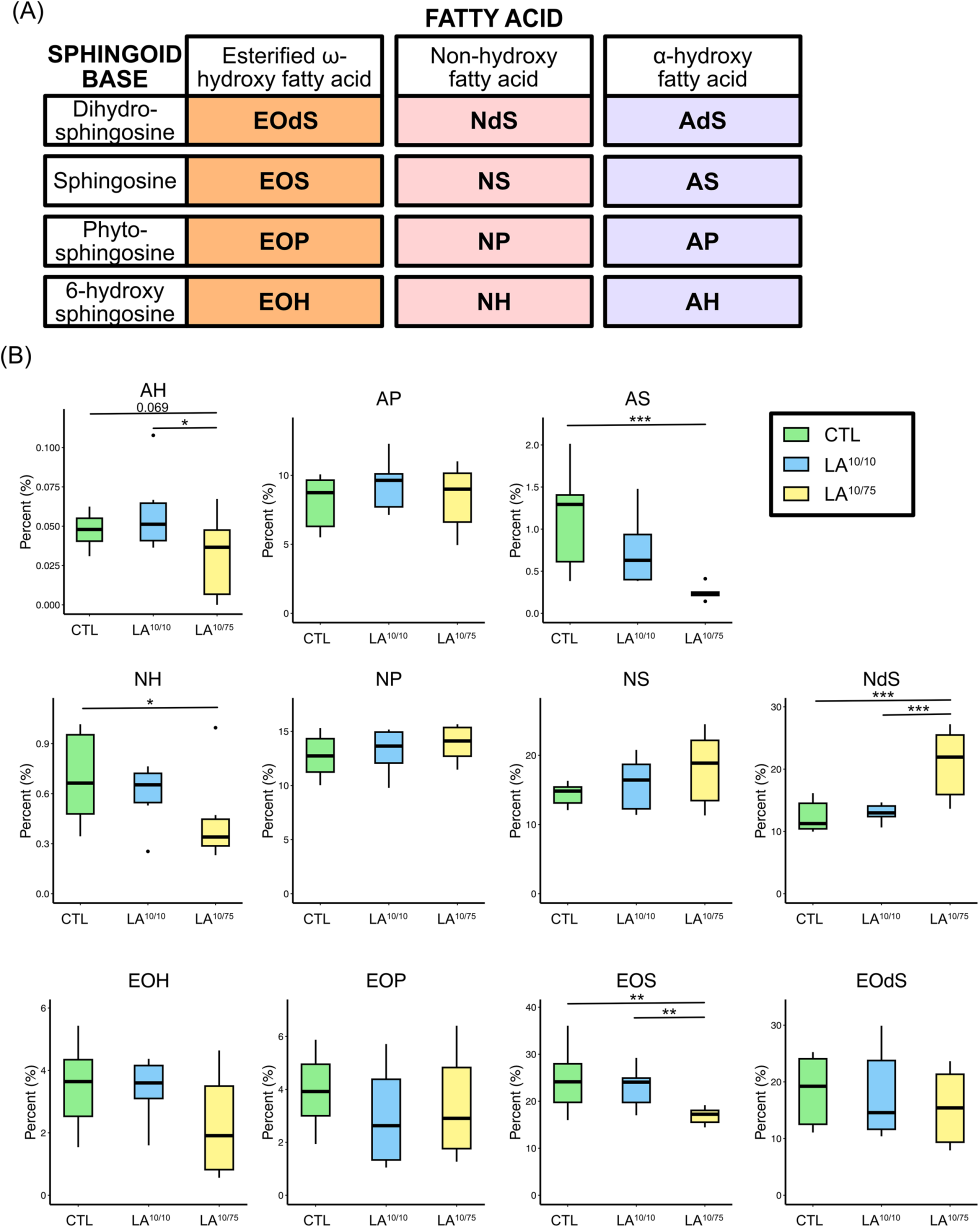
Distribution of ceramide classes in the epidermis of reconstructed skin substitutes. **(A)** Nomenclature of the different classes of ceramides according to their structure and **(B)** the percentage of each ceramide class detected by Lipotype GmbH in the epidermis of skin substitutes across all experimental conditions. The relative abundance of each class was calculated as a percentage of total ceramides identified by Lipotype. Data are presented as median ± minimum and maximum values within 1.5 × the interquartile range (IQR) from the first and third quartiles. *p*-values were derived from a linear mixed-effects model followed by Tukey’s *post hoc* test (N = 3 donors, n=2 skin substitutes per donor). Significance levels: *p-value < 0.05; **p-value < 0.01; ***p-value < 0.001.

Testosterone permeability is markedly reduced in the LA^10/75^ group. This was evidenced by the significantly lower cumulative amounts of testosterone permeating through the skin substitutes from the second hour post-application through the conclusion of the absorption study (**Figure 1M**). The flux values of testosterone can be found in **Table 1**. The only significant decrease for flux values with group LA^10/75^ is during the maximum peak height which is during the fourth timepoint. Moreover, **Supplementary Figure 1** shows significant negative correlations between the flux values and LA proportions in PLs, DAGs and TAGs and the ceramides NdS were observed (for ceramide classes nomenclature, refer to **Figure 2A**). Although no significant changes were observed for the expression of tight junction proteins (**Figures 1N–P**), there was a significant negative correlation between the flux of testosterone and the relative band densities of claudin-1 (CLDN-1). Since CLDN-1 is the tight junction protein known for its role in skin barrier, its function in the skin substitutes appears maintained. Altogether, these correlations indicate that in our model, LA supplementation enhanced the barrier properties of our skin substitutes against testosterone.

**Table 1:** Flux of testosterone through the skin substitutes over 24 hours.

Time point (h)	CTL		LA^10/10^		LA^10/75^	
	Flux ± SEM (μg/cm^2^/h)	p-value	Flux ± SEM (μg/cm^2^/h)	p-value	Flux ± SEM (μg/cm^2^/h)	p-value
1	44 ± 4	ns	51 ± 8	ns	41 ± 4	ns
2	73 ± 6	ns	75 ± 9	ns	61 ± 5	ns
3	95 ± 3	ns	98 ± 6	ns	85 ± 2	ns
4	101 ± 1	*	98 ± 3	ns	94,2 ± 0,7	*
6	75 ± 2	ns	77 ± 3	ns	78,2 ± 0,2	ns
8	45 ± 3	ns	45 ± 5	ns	51 ± 2	ns
24	5,3 ± 0,5	ns	5,0 ± 0,8	ns	6,6 ± 0,8	ns

Significance between flux values at specific timepoints: *p-value 0.05.

### 3.3 High-concentration linoleic acid reduces specific ceramide classes

The major lipid classes in the epidermis of skin substitutes were profiled by untargeted mass spectrometry (Lipotype GmbH). The analysis included ceramide classes (**Figure 2A**), cholesterol esters (CE), DAGs, and TAGs (**Supplementary Figure 3**). Among the ceramide classes, ceramides AS, AH, NH and EOS exhibited a pronounced reduction in the LA^10/75^ group (**Figure 2B**). A consistent reduction in ceramide EOS proportions was observed across most characterized species, with significant decreases noted for ceramides EOS 66:3; O2, 67:3; O2, and 68:3; O2 (**Supplementary Figure 2**). Noteworthy, a general downward trend was observed across most ceramide classes with increasing LA concentration, the ceramide NdS being the only exception, compared to the CTL and LA^10/10^ groups. Ceramides AS distribution were also correlated with the flux of testosterone at the maximum peak timepoint (4h), see **Supplementary Figure 1**.

### 3.4 Linoleic acid supplementation alters fatty acid composition in phospholipids and triacylglycerols

**Figure 3:**
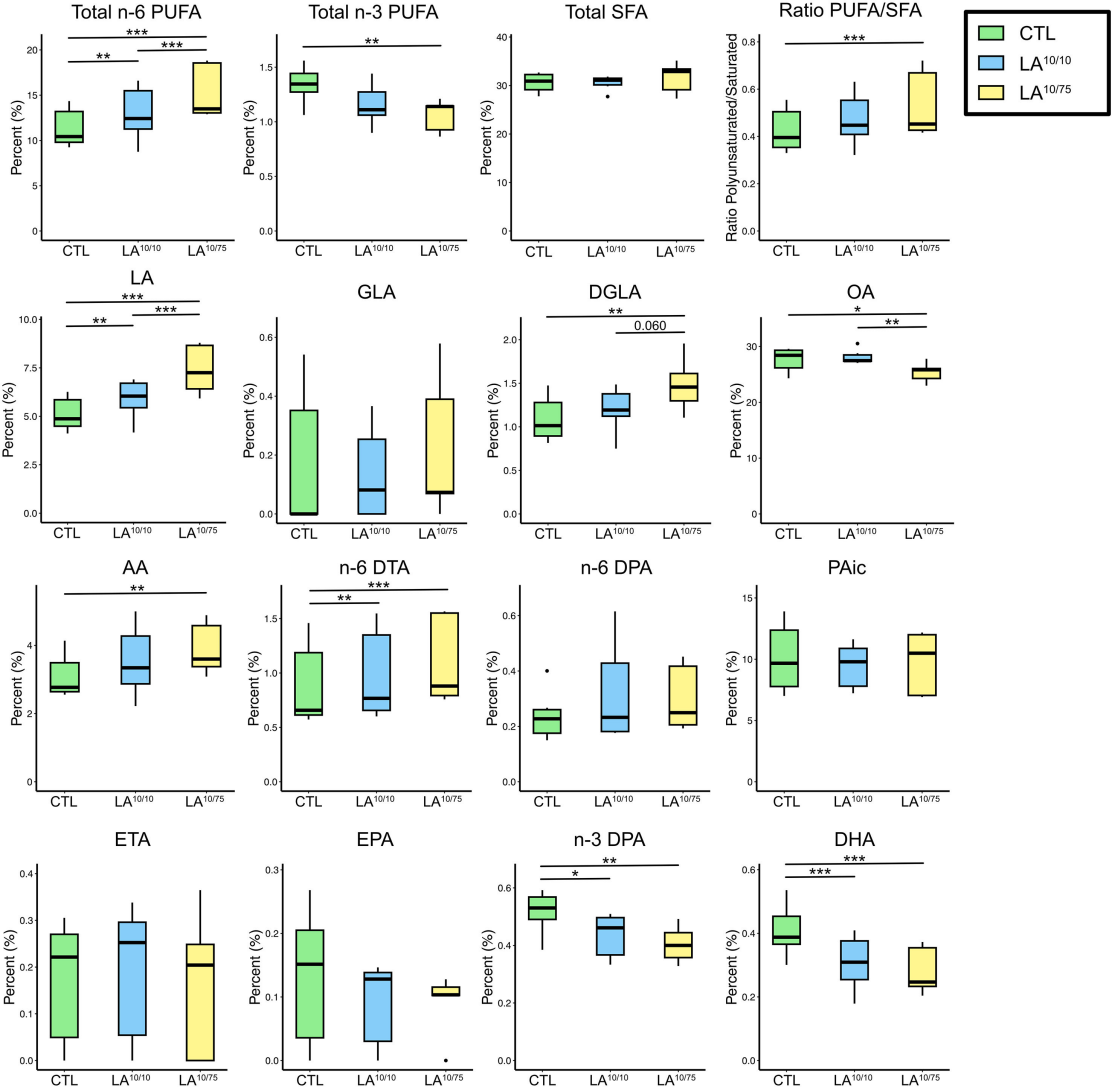
Fatty acid composition of the epidermal phospholipid fraction in reconstructed skin substitutes. Percentage values of fatty acids identified in the phospholipid fraction of the epidermis across all culture conditions. Data are presented as median ± minimum and maximum values within 1.5 × the interquartile range (IQR) from the first and third quartiles. *p*-values were derived from a linear mixed-effects model followed by Tukey’s *post hoc* test (N = 3 donors, n=2 skin substitutes per donor). Significance levels: *p-value < 0.05; **p-value < 0.01; ***p-value < 0.001.

Phospholipidic fatty acid profile was quantified by gas chromatography coupled with mass spectrometry (GC-MS). Total percentage of n-6 polyunsaturated fatty acids (PUFAs) were significantly elevated in the LA^10/75^ group while n-3 PUFA proportions were reduced (**Figure 3**). As expected, LA incorporation into PLs was significantly higher in the LA^10/75^ group than the CTL or LA^10/10^ groups. Among the n-6 PUFAs, dihomo-γ-linolenic acid (DGLA), arachidonic acid (AA), and docosatetraenoic acid (DTA) were also increased in the LA^10/75^ group. In contrast, the n-3 PUFAs, docosapentaenoic acid (DPA) and docosahexaenoic acid (DHA), as well as the monounsaturated fatty acid oleic acid (OA), were decreased following LA supplementation at high concentration during the ALI phase.

**Figure 4:**
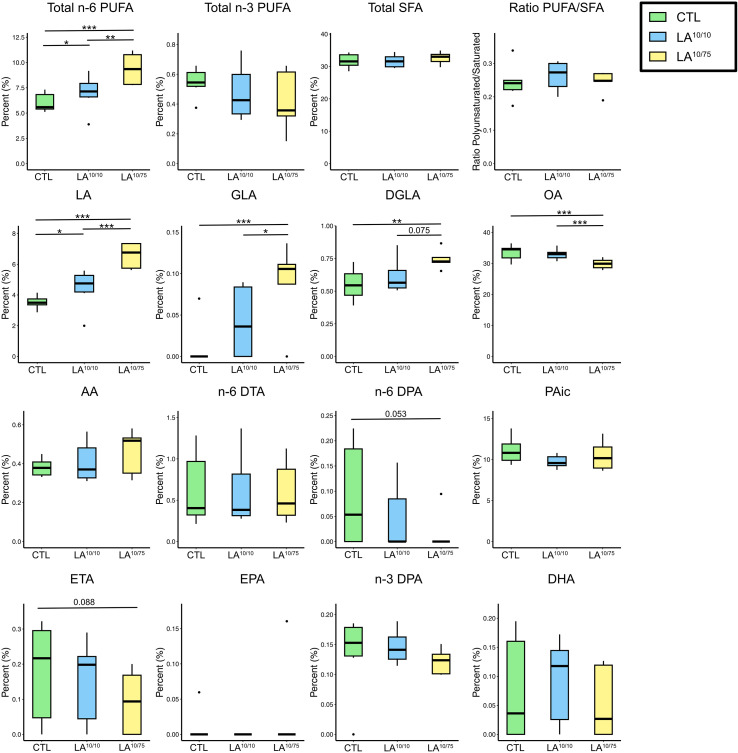
Fatty acid composition of epidermal triacylglycerols in skin substitutes. Percentage values of selected fatty acids identified in the triacylglycerol fraction of the epidermis across all experimental conditions. Data are presented as median ± minimum and maximum values within 1.5 × the interquartile range (IQR) from the first and third quartiles. *p*-values were derived from a linear mixed-effects model followed by Tukey’s *post hoc* test (N = 3 donors, n=2 skin substitutes per donor). Significance levels: *p-value < 0.05; **p-value < 0.01; ***p-value < 0.001.

Total TAG proportions were comparable in our groups (**Supplementary Figure 3**). However, fatty acid composition changed, with an increase in total n-6 PUFAs distribution after a high LA supplementation (**Figure 4**), consistent with the increased incorporation of LA found in phospholipids (**Figure 3**). Indeed, the contents n-6 PUFA in TAGs almost mimicked those found in PLs, except for AA and n-6 DTA, which were not increased in the TAGs of the LA^10/75^ group. γ-linoleic acid (GLA) and n-6 DPA distributions were significantly affected in LA^10/75^ group, with increased GLA and decreased n-6 DPA. Of note, the TAG content in OA also mimicked what was found in PLs while no significant changes were observed for the n-3 PUFAs in any condition, apart from eicosatetraenoic acid (ETA), which was significantly decreased in the LA^10/75^ group.

### 3.5 Linoleic acid supplementation alters diacylglycerol composition

**Figure 5:**
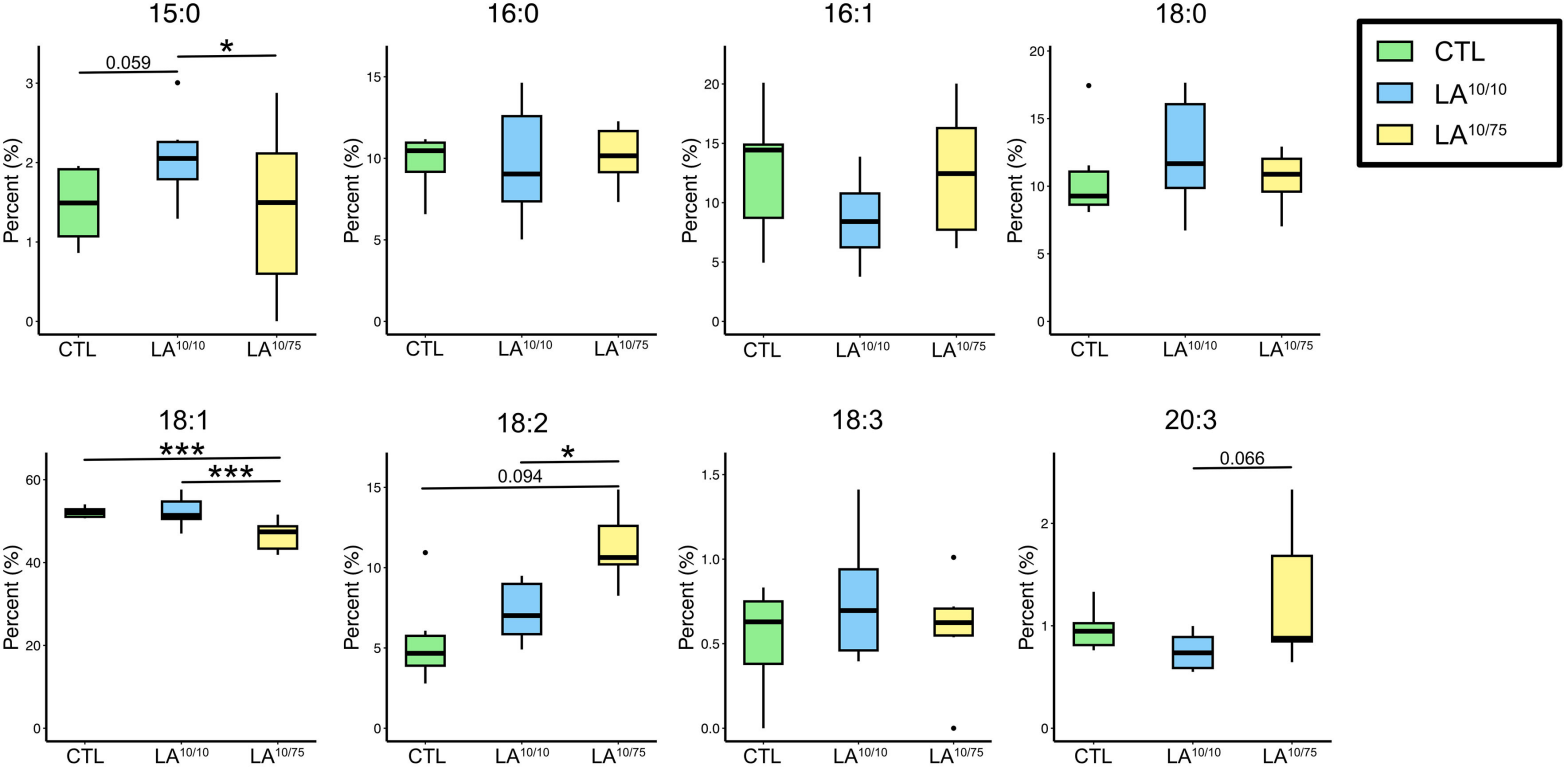
Fatty acid composition of diacylglycerols. Percentage values of selected fatty acids identified in the diacylglycerol fraction of the epidermis across all experimental conditions. Individual fatty acid percentages were calculated based on the relative abundance of each diacylglycerol species. Data are presented as median ± minimum and maximum values within 1.5 × the interquartile range (IQR) from the first and third quartiles. *p*-values were derived from a linear mixed-effects model followed by Tukey’s *post hoc* test (N = 3 donors, n=2 skin substitutes per donor). Significance levels: *p-value < 0.05; ***p-value < 0.001.

Total DAGs proportions were also comparable in our groups (**Supplementary Figure 2**). Given the enhanced incorporation of LA into both TAGs and PLs, the composition of DAGs provides important mechanistic insight. As shown in **Figure 5**, the total fatty acid content of DAGs revealed a significant increase in 18:2 (LA) and 20:3 (DGLA and ALA) in the LA^10/75^ condition. In contrast, a notable decrease in 18:1 (OA and vaccenic acid) and 15:0 (pentadecanoic acid) was observed following high-concentration LA supplementation. These analyses could not resolve double bond localization along the acyl chains, preventing discrimination between GLA and ALA, ETE and DGLA, as well as OA and vaccenic acid; therefore, a simplified nomenclature is used. These findings suggest selective remodeling of DAG species in response to elevated LA availability.

### 3.6 Monoacylglycerol accumulation decreases with high concentration of LA

To investigate the metabolic fate of fatty acids into bioactive lipid mediators, liquid chromatography coupled with tandem mass spectrometry was utilized to quantify oxygenated derivatives and endocannabinoid-like lipids, including MAGs and *N*-acyl-ethanolamines (NAEs). MAGs were found to accumulate in self-assembled skin models relative to NHS, a phenomenon further amplified by fatty acid supplementation (Simard et al., 2021). Herein, a constant supplementation with 10 μM LA (LA^10/10^) significantly increased levels of 1/2-linoleoylglycerol (1/2-LG) and 1/2-oleoylglycerol (1/2-OG) compared to CTLs. Curiously, increasing the concentration of LA during the ALI phase did not impact their levels compared to CTLs and the levels of 1/2-OG were even significantly decreased compared with LA^10/10^. Among all detected lipid mediator classes, MAGs exhibited the most significant alterations (see **Supplementary Table 3**).

### 3.7 Main bioactive lipid mediators found in the skin model

Surprisingly, the levels of LA itself, while trending to be higher, were not statistically different in the LA-enriched conditions. In addition, the metabolism of LA into oxygenated products was assessed. While levels of 9-hydroxyoctadecadienoic acid (9-HODE) and 13-hydroxyoctadecadienoic acid (13-HODE) trended upward in the LA^10/75^ group, only the 13-HODE-derived *N*-acyl-ethanolamine (13-HODE-EA) and the 13-HODE-derived-glycerol (13-HODE-G) reached statistical significance. These findings highlight the complex and dose-sensitive modulation of bioactive lipid mediator profiles by LA in reconstructed skin models.

### 3.8 Linoleic acid alters the expression of lipid-metabolizing enzymes

**Figure 6:**
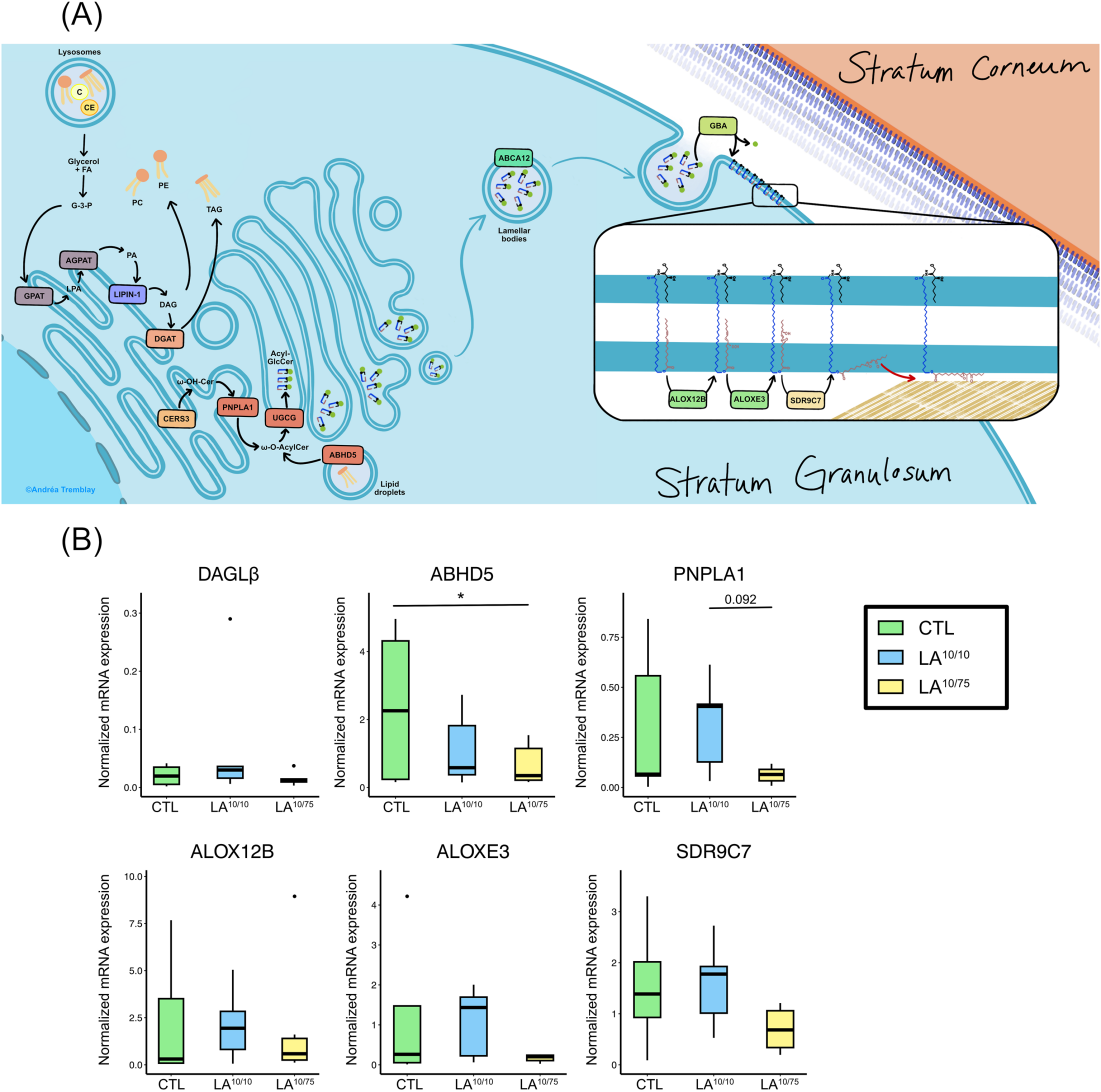
Lipid metabolism enzymes in the formation of the corneocyte lipid envelope (CLE). **(A)** The CLE forms as keratinocytes undergo terminal differentiation. Ceramides are synthesized in the endoplasmic reticulum (ER), while ω-O-acylceramides are produced by PNPLA1 following its recruitment by ABHD5. These specialized lipids are stored in lamellar bodies and transported to the stratum corneum (SC). Upon reaching the SC, lamellar body contents are extruded, and the linoleate moiety of ω-O-acylceramides serves as a substrate for the lipoxygenases ALOX12B and ALOXE3. Subsequent oxidation by SDR9C7 generates a reactive epoxy-enone intermediate capable of covalently binding to the corneocyte envelope, contributing to CLE formation. **(B)** mRNA expression of selected lipid metabolism enzymes normalized to GAPDH across all experimental conditions. Data are presented as median ± minimum and maximum values within 1.5 × the interquartile range (IQR) from the first and third quartiles. *p*-values were derived from a linear mixed-effects model followed by Tukey’s *post hoc* test (N = 3 donors, n=2 skin substitutes per donor). Significance levels: * *p*-value < 0.05.

Given the observed reduction in ceramide EOS proportions following high-concentration LA supplementation (**Figure 2**) and the concurrent improvement in barrier function in percutaneous absorption studies, we investigated the expression of key lipid-metabolizing enzymes to better understand the underlying lipidomic shifts (**Figure 6A**). Gene expression analysis was performed by RT-qPCR for enzymes involved in LA release from TAGs and DAGs (Abhydrolase Domain Containing 5, Lysophosphatidic Acid Acyltransferase (ABHD5), patatin-like phospholipase domain-containing-1 (PNPLA1), diacyglycerol lipase β (DAGLβ)), and CLE formation (arachidonate 12-lipoxygenase, 12R type (ALOX12B), epidermis-type lipoxygenase 3 (ALOXE3), dehydrogenase/reductase family 9C member 7 (SDR9C7)) (**Figure 6B**).

**Figure 7:**
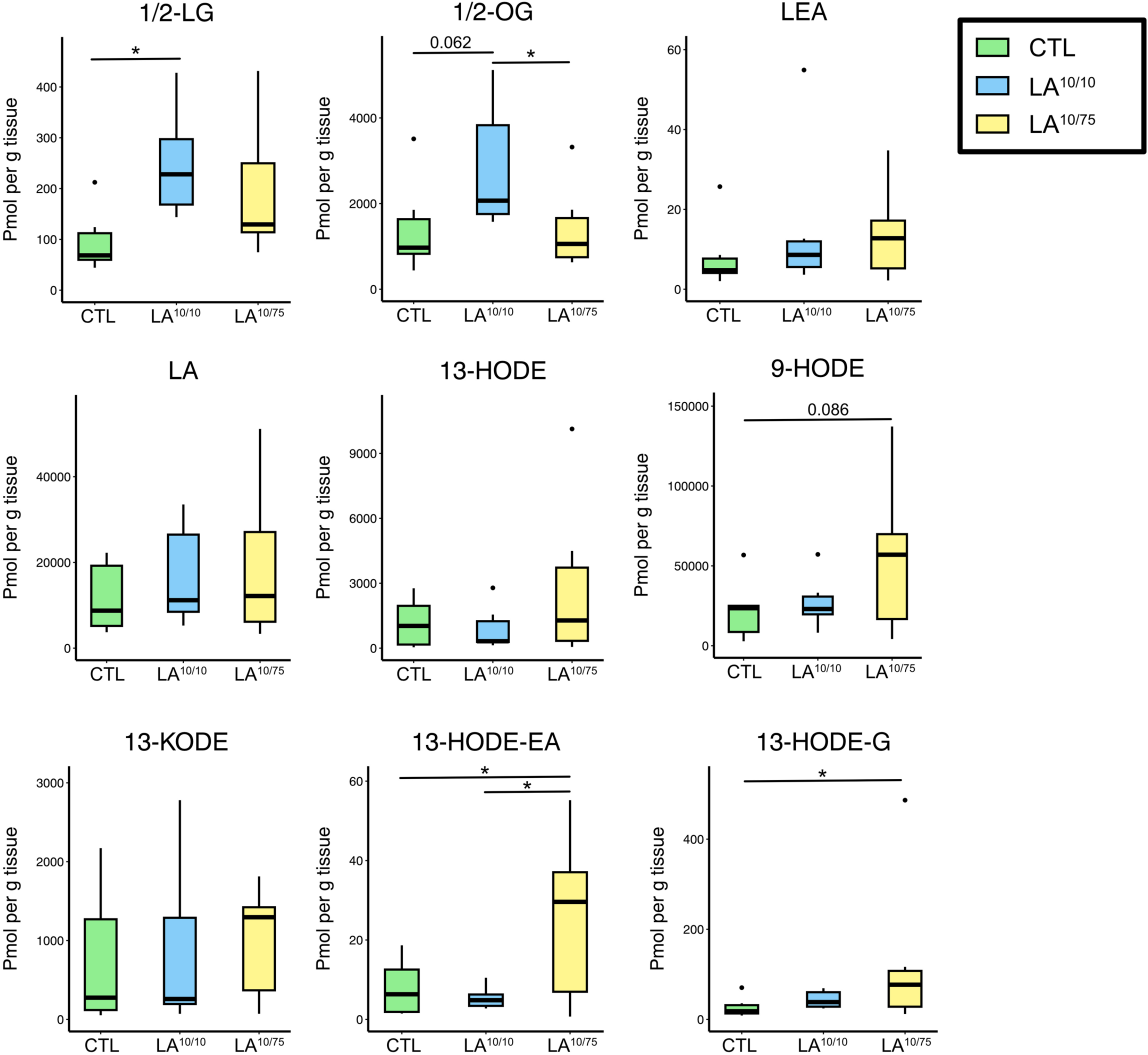
LA-derived lipid mediators and oleic acid-containing monoacylglycerol in the epidermis of skin substitutes. Levels of selected linoleic acid-derived lipid mediators and monoacylglycerol containing oleic acid, expressed in pmol per gram of epidermal tissue, across all experimental conditions. Data are presented as median ± minimum and maximum values within 1.5 × the interquartile range (IQR) from the first and third quartiles. Significance levels: *p-value < 0.05. p-values were derived from a linear mixed-effects model followed by Tukey’s post hoc test (N = 3 donors, n=2 skin substitutes per donor).

Expression of ABHD5 was significantly reduced in the LA^10/75^ group compared to non-supplemented controls. Additionally, PNPLA1 and SDR9C7 expression trended to be decreased in the LA^10/75^ group, compared to those supplemented with 10 μM LA. However, no significant changes were observed for ALOX12B, ALOXE3, PNPLA1 and SDR9C7. The ABHD5 transcriptional changes may contribute to the altered ceramide profile under high LA supplementation. The expression of DAGLβ was similar across all culture conditions despite the modulated levels of MAGs found between 10 μM supplemented substitutes and the other groups seen in **Figure 7**.

## 4 Discussion

This study demonstrates that LA was efficiently incorporated into major lipid classes, including PLs, TAGs, and DAGs, with the exception of MAGs, which were abnormally elevated in self-assembled skin models (Simard et al., 2021). Interestingly, high-concentration LA normalized MAG levels, aligning them more closely with those of non-supplemented controls. Functionally, LA supplementation improved skin barrier performance, as evidenced by reduced testosterone permeability in **Figure 1**. This suggests that LA contributes not only to structural lipid integration but also to functional enhancement of the barrier. The reduction in testosterone permeability was modest, which is not unexpected given that testosterone is highly lipophilic and therefore only partially reflects overall barrier competence. Indeed, alterations in barrier function often result in relatively limited changes in its permeation compared to more hydrophilic compounds (Tsai et al., 2001).

As previously noted, CLDN-1 protein expression was not significantly altered by either LA supplementation condition. However, correlation analysis presented in **Supplementary Figure 1** shows that CLDN-1 expression increases as testosterone flux decreases across the skin substitutes, supporting its functional relevance in the self-assembled model. This is consistent with previous studies linking reduced CLDN-1 levels to impaired barrier function (Tokumasu et al., 2016; Bergmann et al., 2020). Notably, CLDN-1 expression also positively correlates with LA incorporation into TAGs, suggesting a potential relationship between lipid remodeling and tight junction integrity, which was previously demonstrated (Tremblay et al., 2021). The correlation matrix further indicates that the only other variable associated with reduced testosterone flux at hour 4, aside from CLDN-1, is the level of LA incorporated into TAGs. This observation is particularly relevant given prior findings that topical application of LA and GLA-containing TAGs to essential fatty acid-deficient rats significantly reduced transepidermal water loss (TEWL), thus improving skin barrier (Hartop and Prottey, 1976). Together, these results suggest that LA-enriched TAGs may contribute to barrier reinforcement through both lipid matrix and junctional protein modulation.

Notably, ceramide AS proportions were significantly reduced in the LA^10/75^ group (**Figure 2B**). This reduction showed an inverse correlation with CLDN-1 expression, as well as with LA content in both TAGs and PLs. Ceramides AH and NH proportions were also significantly decreased in the epidermis of the LA^10/75^ group compared with controls, however no associations were observed with tight junction protein expression or LA incorporation into epidermal lipids. In contrast, ceramide NdS proportions were significantly increased in the LA^10/75^ group (**Figure 2B**). In adipocytes, conjugated-LA has been shown to increase ceramide levels while reducing TAG content compared with control LA supplementations, highlighting that sphingolipid metabolism can be regulated independently from neutral lipid storage (Wang and Fromm, 2015). In the present study, no significant changes were observed for ceramides containing a phytosphingosine base. Interestingly, sphingosine-based ceramides have been reported to exhibit higher permeability than their phytosphingosine counterparts, suggesting that variations in ceramide backbone composition may further contribute to differences in barrier function (Uche et al., 2019a).

Unexpectedly, ceramide EOS proportions were reduced in the substitutes LA^10/75^, despite improved barrier function. One hypothesis was that more ceramide EOS might be incorporated into the CLE, enhancing barrier integrity. However, gene expression analysis (**Figure 6**) revealed significant downregulation of ABHD5 and a downward trend in PNPLA1, enzymes essential for ω-O-acylceramide synthesis. ABHD5 recruits PNPLA1 to lipid droplets, enabling esterification of ceramides with LA (Kien et al., 2018). These findings suggest that reduced ceramide EOS percentage stems from impaired synthesis, rather than enhanced incorporation into the CLE.

We also examined the expression of ALOX12B, ALOXE3, and SDR9C7, which are involved in the final steps of CLE formation. Previous studies have in fact established that knock out mice of ALOX12B and ALOXE3 had impaired skin barrier but also decreased protein-bound ceramide levels (Krieg et al., 2013). Other studies focused their research on the role of SDR9C7 in the skin and have shown that a knock out mice of SDR9C7 significantly decreased levels of covalently bound ceramides OS in the skin, in addition to having increased TEWL results (Takeichi et al., 2020). In our study, their expression showed no significant changes but trended downward in substitutes LA^10/75^, reinforcing the hypothesis that decreased synthesis via ABHD5 and PNPLA1 is the primary mechanism.

Importantly, these transcriptional changes do not necessarily indicate impaired barrier formation. In contrast, the reduced ceramide EOS levels observed in the LA^10/75^ condition may instead reflect a remodeling of epidermal lipid metabolism and altered partitioning of LA between TAGs and ceramide synthesis pathways. To better contextualize these transcriptional changes, a public single-cell RNA-seq dataset of human epidermis was analyzed and keratinocyte differentiation states were associated with the expression of lipid-metabolizing enzymes (**Supplementary Figure 4** and related supplementary method) (He et al., 2020). These analyses showed that enzymes involved in ceramide EOS processing and CLE formation, including PNPLA1, ALOX12B, ALOXE3 and SDR9C7, were predominantly associated with more differentiated keratinocyte populations.

Interestingly, ABHD5, the only significantly downregulated gene in LA^10/75^, showed a non-layer-specific expression profile in the single-cell dataset. Given its role in mobilizing TAG-associated lipid droplets for ceramide EOS synthesis, its reduction is consistent both with the lower ceramide EOS proportions and the accumulation of LA within TAG species observed in this condition. Indeed, knockout mice for ABHD5 and PNPLA1 both exhibited reduced levels of ceramides EOS, and ABHD5 knockout additionally resulted in a marked accumulation of TAGs, consistent with the present study (Hirabayashi et al., 2017). Overall, these results further support the complex relationship between keratinocyte differentiation, epidermal lipid remodeling, and barrier function in reconstructed skin models.

Oxylipins such as 9-HODE were detectable across all groups. Interestingly, levels of 9-HODE tended to increase in substitutes LA^10/75^ compared to other conditions. This contrasts with unchanged ALOX12B transcript levels in **Figure 6**. Moreover 9-HODE is predominantly generated via hydrolysis of LOX-oxidized ceramides (Siebert et al., 2001; Tyrrell et al., 2021; Noguchi et al., 2023). Although 9-HODE is associated with pro-inflammatory signaling in the skin, previous work using the same model reported levels in control substitutes comparable to NHS (Simard et al., 2021). In the present study, 9-HODE levels in the LA^10/75^ condition remained within the same range as those reported for NHS, suggesting that the model is not overtly inflamed (Simard et al., 2021). To investigate the production of non-enzymatic 9-HODE, chiral chromatography analyses would have allowed identification of the difference between 9(R)-HODE and 9(S)-HODE, which would have helped determine its origin. Also, the possibility that 9-HODE does not arise from oxidized ceramides and is rather the consequence of autoxidation or an oxygenase cannot be excluded (Laneuville et al., 1995; Jira and Spiteller, 1999).

A previous *in vivo* study showed that ALOX12B knockout mice had modulated levels of ester-linked epidermal lipids, despite unchanged total free ceramides and fatty acids and although its deletion caused a lethal phenotype due to a barrier function defect, tight junction proteins were not affected (Epp et al., 2007).

An intriguing observation in our study was the consistent and significant decrease in OA proportions across all lipid classes following high-LA supplementation. This trend is particularly noteworthy given OA’s known capacity to substitute for LA under conditions of deficiency, often at the expense of barrier integrity (Vičanová et al., 1999). The decline in OA concurrent with elevated LA incorporation suggests a preferential utilization of LA when available, potentially restoring lipid composition closer to that of native epidermis and supporting the formation of a more physiologically relevant stratum corneum.

While increased LA levels were documented in selected lipid species, acyltransferases, elongases, and other phospholipid-remodeling enzymes were not further investigated. Nevertheless, the fatty acid composition of LA-enriched samples shows elevated levels of n-6 PUFAs, such as DGLA and AA, strongly supporting the elongation and desaturation of the supplemented LA. Further investigation will be required to determine whether LA itself can modulate the expression of these enzymes, an aspect that warrants future study.

## 5 Conclusion

Taken together, our results reveal a complex interplay between LA supplementation, lipid remodeling, and enzymatic regulation. The improved barrier function appears to result from coordinated changes across lipid classes and bioactive lipid mediators, rather than a single lipid species. While our lipidomic data provides strong evidence for LA incorporation into structural and signaling lipids, the precise localization and functional integration of these lipids within the epidermal architecture remain to be fully elucidated.

A key limitation of this study is the absence of direct quantification of protein-bound ceramides, which would be necessary to confirm their contribution to CLE formation. Future studies should employ imaging-based or biochemical techniques to assess CLE structure and composition. Such analyses would provide a more comprehensive understanding of the structural and functional changes induced by LA supplementation in reconstructed skin models.

## Data Availability

The raw data supporting the conclusions of this article will be made available by the authors, without undue reservation.

## Glossary

CLDN-1: claudin-1
CTL: control
LA: linoleic acid
LOR: loricrin
OCLN: occludin
SC: stratum corneum
TGM-1: transglutaminase-1
VE: viable epidermis
ZO-1: zonula occludens-1
ns: not significant
SEM: standard error of the mean
A: α-hydroxy fatty acid
dS: dihydrosphingosine
EO: esterified ω-hydroxy fatty acid
H: 6-hydroxy sphingosine
N: non-hydroxy fatty acid
P: phytosphingosine
S: sphingosine
AA: arachidonic acid
DGLA: dihomo-γ-linoleic acid
DHA: docosahexaenoic acid
DPA: docosapentaenoic acid
DTA: docosatetraenoic acid
EPA: eicosapentaenoic acid
ETA: eicosatetraenoic acid
GLA: γ-linoleic acid
n-3: ω-3
n-6: ω-6
OA: oleic acid
PAic: palmitoleic acid
PUFA: polyunsaturated fatty acids
SFA: saturated fatty acids
AA: arachidonic acid
1/2-LG: 1/2-linoleoyl glycerol
1/2-OG: oleoyl glycerol
9-HODE: 9-hydroxyoctadecadienoic acid
13-HODE: 13- hydroxyoctadecadienoic acid
13-HODE-EA: N-13-hydroxyoctadecadienoyl-ethanolamine
13-HODE-G: 13-hydroxyoctadecadienoyl glycerol
13-KODE: 13-keto-9,11-octadecadienoic acid
LEA: N-linoleoyl-ethanolamine
ABCA12: ATP binding cassette subfamily A member 12
ABHD5: abhydrolase domain containing 5, lysophosphatidic acid acyltransferase
Acyl-GlcCer: acylglucosylceramide
AGPAT: 1-acylglycerol-3-phosphate o-acyltransferase
ALOX12B: arachidonate 12-lipoxygenase, 12R type
ALOXE3: epidermis-type lipoxygenase 3
CERS3: ceramide synthases 3
CTL: control
DAG: diacylglycerol
DAGLβ: diacylglycerol lipase β
DGAT: diacylglycerol acyltransferase
FA: fatty acid
G-3-P: glyceraldehyde 3-phosphate
GBA: β-glucocerebrosidase
GPAT: glycerol-3-phosphate acyltransferase
LPA: lysophosphatidic acid
PA: phosphatidic acid
PC: phosphatidylcholine
PE: phosphatidylethanolamine
PNPLA1: patatin-like phospholipase domain-containing-1
SDR9C7: dehydrogenase/reductase family 9C member 7
TAG: triacylglycerol
UGCG: UDP-glucose ceramide glucosyltransferase
ω-O-AcylCer: ω-O-acylceramide
ω-OH-Cer: ω-hydroxy ceramide
