# Supplementary material for: Linoleic acid concentration at the air-liquid interface is key for optimal incorporation into skin model lipidome
Source: Front Physiol. 2026 Jun 22;17:1838020. doi: 10.3389/fphys.2026.1838020 (PMC13334480; doi:10.3389/fphys.2026.1838020)
Supplement: Supplementary file 1 [file DataSheet1.docx]

Supplementary Material

**Supplementary method**

**Single-cell RNA-sequencing analysis**

Single-cell transcriptomic data were obtained from the GSE147424 dataset published by He and colleagues in 2020 (He et al., 2020). In this dataset, biopsy specimens were converted into single-cell suspensions and subjected to droplet-based scRNA-seq using the 10x Genomics platform. To limit biological variability and focus on baseline skin homeostasis, only healthy samples were included. Eight samples were selected from GEO, including GSM4430462, GSM4430464, GSM4430466, GSM4430467, GSM4430468, GSM4430470, GSM4430471, and GSM4430475.

Raw expression matrices were imported into Seurat for analysis. Quality control filtering excluded cells with fewer than 100 or more than 5000 detected genes, as well as cells with mitochondrial content above 25 percent. Ribosomal genes, long non-coding RNAs, microRNAs, and small nucleolar RNAs were also removed. These criteria followed the original study (He et al., 2020).

Data were normalized using the LogNormalize method with a scaling factor of 10000, and highly variable genes were identified using the variance-stabilizing transformation (VST) approach. Batch effects were corrected using reciprocal principal component analysis (RPCA) based integration. PCA was then performed, and the first 20 components were used for neighbor graph construction, Louvain clustering at a resolution of 0.4, and UMAP embedding. UMAP parameters were optimized using dimensions 1 to 10, a neighbor value of 25, and a minimum distance of 0.5.

Keratinocyte clusters were annotated based on canonical markers (KRT5, KRT14, KRT1 and KRT10). Expression of genes involved in ceramide metabolism and CLE formation was analyzed and visualized on global UMAP embeddings. Gene expression values were scaled between 0 and 1.

## Supplementary Figures

**
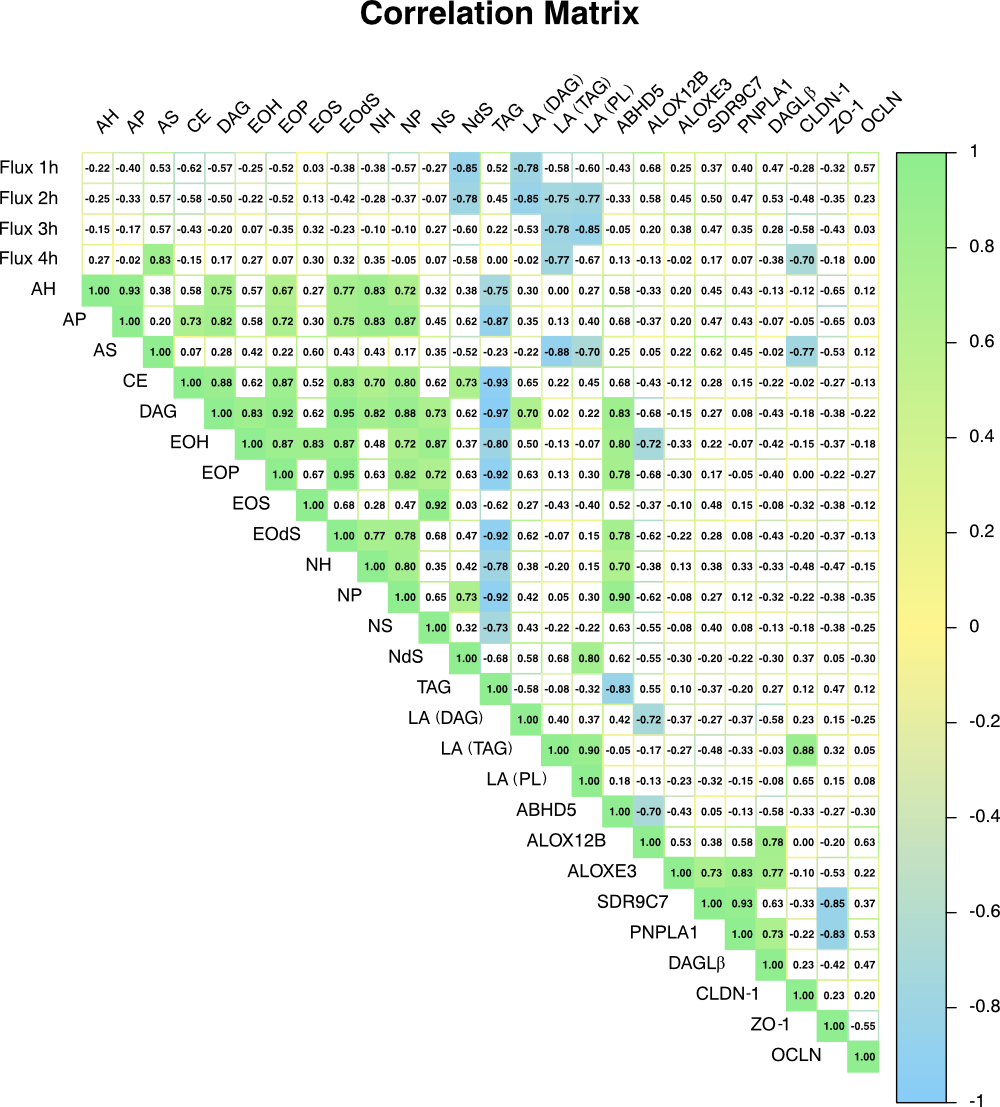
**

**Figure S1. Spearman correlation matrix of variables presented in the study.** Pairwise Spearman correlations were calculated using complete observations across key parameters, including lipid species, gene expression levels, and skin barrier metrics (testosterone flux and tight junction expression). Significant correlations (p < 0.05) were color-coded: green for positive and blue for negative correlations. The matrix provides an overview of interdependencies among measured variables.


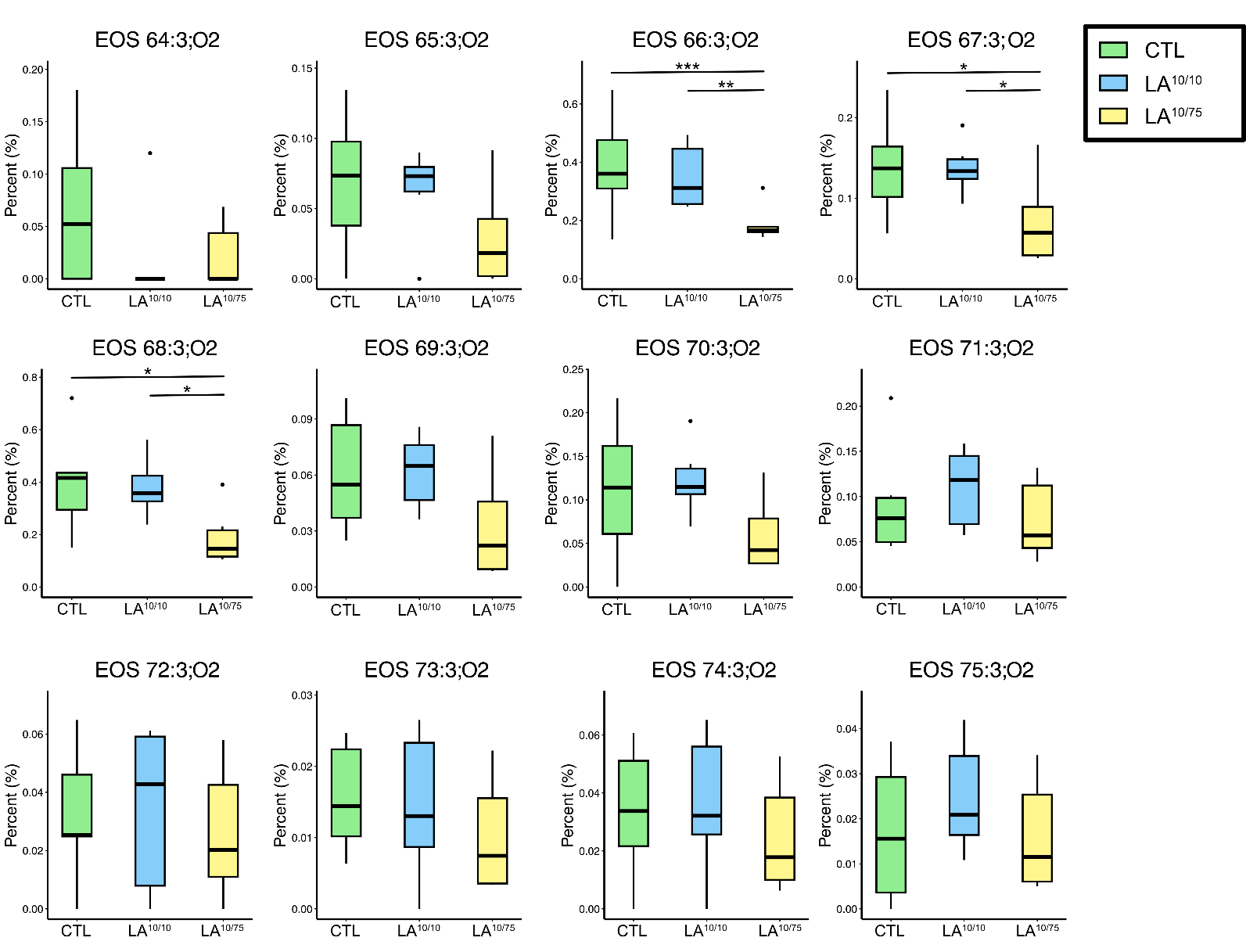


**Figure S2. Distribution of ω-hydroxy-sphingosine (EOS) ceramide species in the epidermis.** The percentage of each ceramide EOS species detected by Lipotype GmbH in the epidermis of skin substitutes across all experimental conditions. The relative abundance of each species was calculated as a percentage of total lipid identified by Lipotype. Data are presented as median ± minimum and maximum values within 1.5 × the interquartile range (IQR) from the first and third quartiles. p-values were derived from a linear mixed-effects model followed by Tukey’s post hoc test (N=3 donors, n=2 skin substitutes per donor). Abbreviations: CTL, control; LA, linoleic acid. Significance levels: * p-value < 0.05; ** p-value < 0.01; *** p-value < 0.001.

**
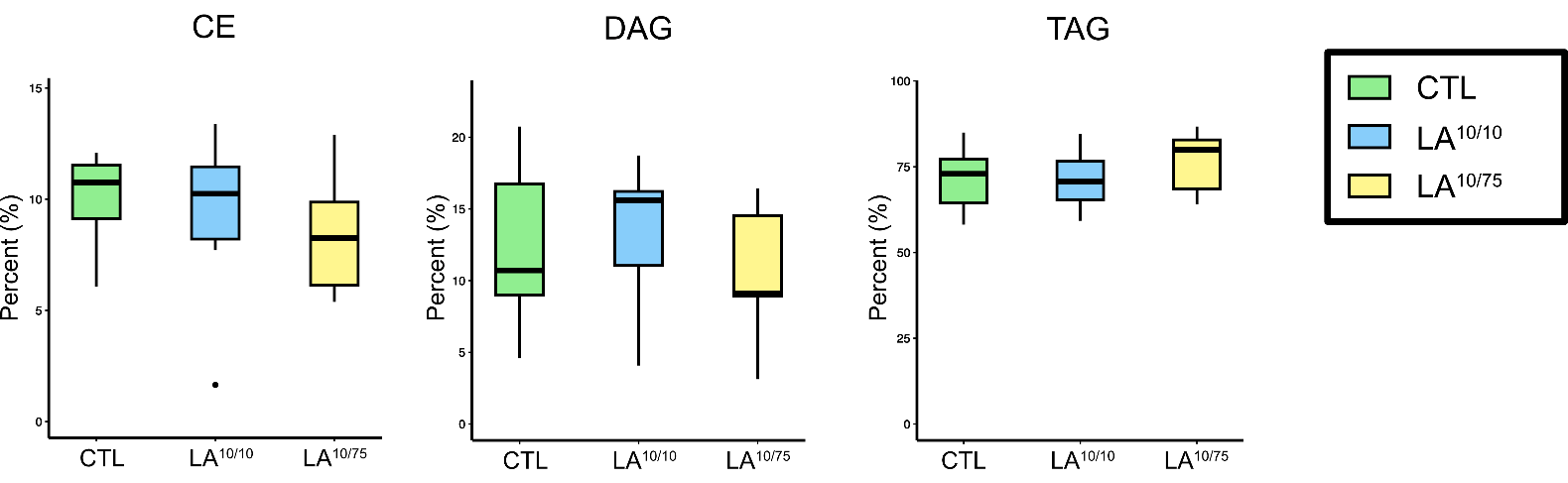
**

**Figure S3. Distribution of the most abundant lipid classes in the epidermis of reconstructed skin substitutes.** The percentage of cholesterol ester (CE), diacylglycerol (DAG) and triacylglycerol (TAG) detected by Lipotype GmbH in the epidermis of skin substitutes across all experimental conditions. The relative abundance of each class was calculated as a percentage of total lipid identified by Lipotype. Data are presented as median ± minimum and maximum values within 1.5 × the interquartile range (IQR) from the first and third quartiles. p-values were derived from a linear mixed-effects model followed by Tukey’s post hoc test (N=3 donors, n=2 skin substitutes per donor). Abbreviations: CTL, control; LA, linoleic acid.

**
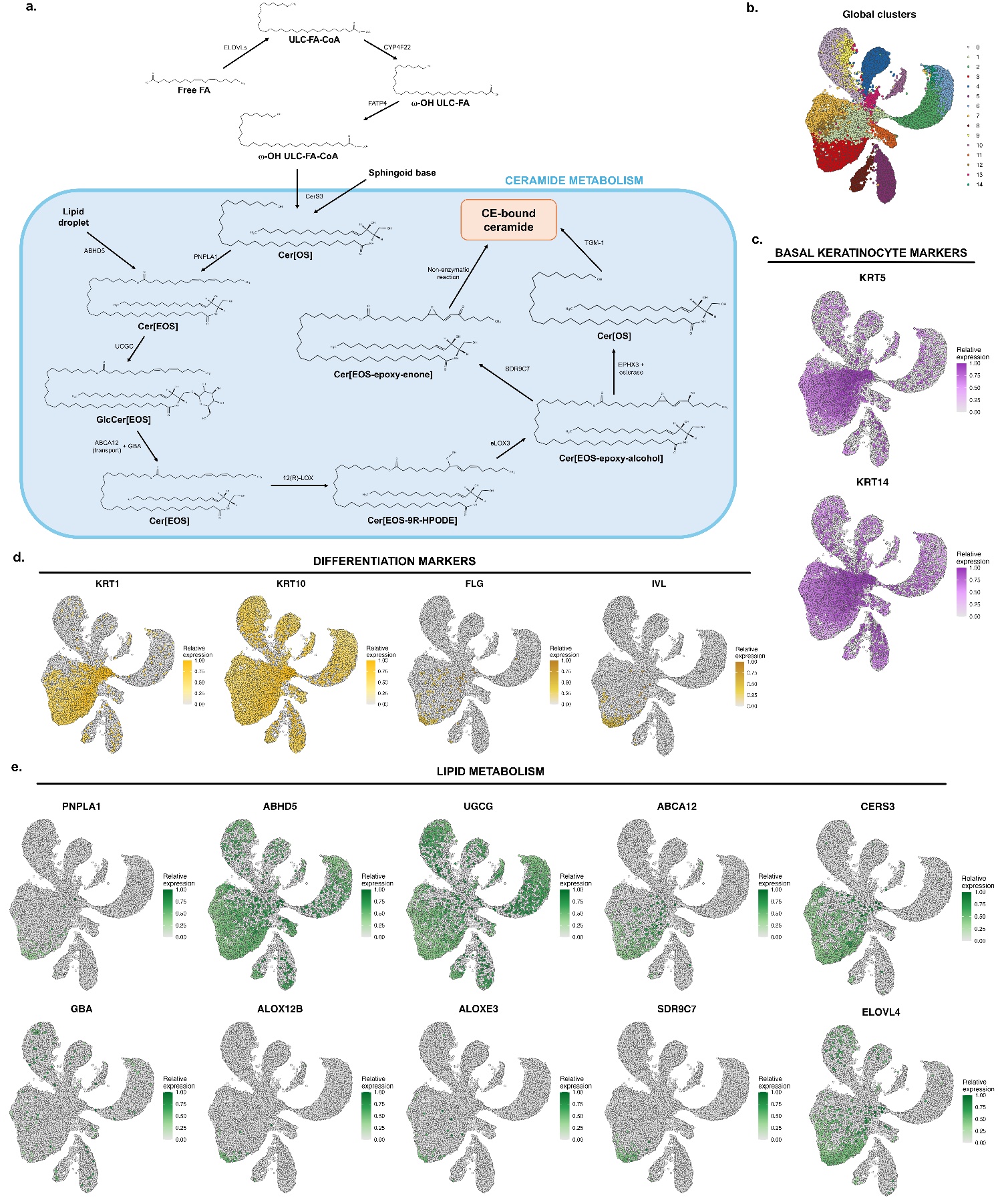
**

**Figure S4. Stratification of enzymes involved in ceramide synthesis and cornified lipid envelope formation in a human keratinocyte subset.** (a) Schematic representation of ceramide synthesis and metabolism in the skin. (b) UMAP plot of integrated skin cells and cluster identification. (c) UMAP plot of reintegrated keratinocyte subset for stratification (d) according to their differentiation state. (e) UMAP of relative cellular distribution of each gene associated with the studied ceramide metabolism. Abbreviations: ABCA12, ATP binding cassette subfamily A member 12; ABHD5, abhydrolase domain containing 5, lysophosphatidic acid acyltransferase; ALOX12B, arachidonate 12-lipoxygenase, 12R type; ALOXE3, epidermis-type lipoxygenase 3; CERS3, ceramide synthase 3; ELOVL, very-long-chain fatty acid elongase; FLG, Filaggrin; GBA, β-glucocerebrosidase; IVL, involucrin; KRT, keratin; PNPLA1, patatin-like phospholipase domain-containing-1; SDR9C7, dehydrogenase/ reductase family 9C member 7; UGCG, UDP-glucose ceramide glucosyltransferase.

**Table S1. Mean levels of lipid mediator classes found in the epidermis of skin substitutes.**

| Lipid mediator | Mean levels of lipid mediators  (pmol per g tissue) | | |
| --- | --- | --- | --- |
|  | CTL | LA^10/10^ | LA^10/75^ |
| 6-keto PGF1a | 16,86 | 31,55 | 7,93 |
| PGF3a | 0,00 | 0,00 | 0,00 |
| TXB2 | 0,00 | 0,00 | 0,00 |
| PGE3 | 1,37 | 1,17 | 1,77 |
| PGF2a | 7,43 | 7,06 | 13,77 |
| PGE1 | 5,22 | 5,71 | 27,03 |
| PGE2 | 42,61 | 49,09 | 96,92 |
| PGD2 | 0,00 | 0,00 | 0,00 |
| 1a, 1b-dihomo PF2a | 0,77 | 0,92 | 0,35 |
| 12(S)-HHTrE | 1,33 ^a^ | 1,84 | 4,35 ^a^ |
| PGF2a-EA | 0,24 | 0,19 | 0,47 |
| PGE2-EA | 0,00 | 0,00 | 0,00 |
| PGD2-EA | 0,00 | 0,10 | 0,15 |
| PGF2a-G | 0,00 | 0,00 | 0,00 |
| PGE2-G | 0,01 | 0,01 | 0,00 |
| PGD2-G | 0,00 | 0,00 | 0,00 |
| SDEA | 0,00 | 0,00 | 0,00 |
| EPEA | 0,11 | 0,12 | 0,08 |
| LEA | 8,29 | 15,57 | 13,98 |
| DHEA | 0,14 | 0,18 | 0,22 |
| AEA | 3,60 | 8,19 | 4,42 |
| PEA | 20,62 | 47,16 | 55,82 |
| OEA | 243,39 | 540,40 | 410,85 |
| SEA | 413,13 ^b^ | 1134,70 ^b^ | 1164,48 |
| 1/2-SDG | 0,00 | 0,00 | 0,00 |
| 1/2-EPG | 0,37 ^b^ | 0,96 ^b / ccc^ | 0,14 ^ccc^ |
| 1/2-DHG | 5,67 | 9,76 | 6,31 |
| 1/2-LG | 96,20 ^b^ | 249,49 ^b^ | 193,97 |
| 1/2-AG | 58,63 | 121,28 | 103,92 |
| 1/2-DPG | 19,60 ^b^ | 34,80 ^b / cc^ | 22,18 ^cc^ |
| 1/2-OG | 1421,51 | 2816,64 ^c^ | 1428,89 ^c^ |
| 1/2-PG | 3029,54 | 2413,52 | 7340,38 |
| SDA | 0,00 ^b^ | 7,76 ^b / c^ | 0,00 ^c^ |
| DPEA(n-6) | 0,03 ^b^ | 0,08 ^b / cc^ | 0,02 ^cc^ |
| DPEA(n-3) | 0,83 | 1,46 | 0,33 |
| EPA | 229,44 ^b^ | 398,91 ^b / cc^ | 190,45 ^cc^ |
| DHA | 353,97 | 605,05 | 300,22 |
| AA | 2376,39 | 4299,53 | 4097,76 |
| DPA (n-3) | 1004,71 | 1929,17 | 776,02 |
| DPA (n-6) | 194,56 | 411,66 | 187,91 |
| LA | 11703,81 | 16726,15 | 19177,21 |
| OA | 21036,25 | 19810,60 | 19240,81 |
| 15-HEPE-EA | 0,11 | 0,09 | 0,17 |
| 13-HODE-EA | 7,97 ^aa^ | 5,43 ^cc^ | 25,68 ^aa / cc^ |
| 15-HETE-EA | 0,00 | 0,00 | 0,19 |
| 15-HEPE-G | 0,58 | 0,19 | 0,00 |
| 12-HEPE-G | 0,00 | 0,67 | 3,19 |
| 13-HODE-G | 27,14 ^a^ | 43,80 | 130,38 ^a^ |
| 17-HDPA-EA | 0,28 | 0,12 | 0,29 |
| 15-HETE-G | 14,73 | 21,27 | 41,61 |
| 17-HDHA-EA | 0,64 | 0,00 | 0,00 |
| 17-HDPA-G | 0,00 | 0,00 | 0,00 |
| 5-HETE-EA | 0,00 | 0,00 | 0,00 |
| 5-KETE-EA | 0,00 | 0,00 | 0,00 |
| 5-HETE-G | 0,00 | 0,00 | 0,00 |
| 5-KETE-G | 0,00 | 0,00 | 0,00 |
| 18-HEPE | 5,00 | 4,32 | 3,62 |
| 8(S),15(S)-DiHETE | 26,95 | 29,47 | 41,75 |
| 5(S),15(S)-DiHETE | 24,67 | 15,20 | 22,79 |
| 5(S),6(R)-DiHETE | 0,00 | 0,00 | 0,47 |
| 13(S)-HOTrE | 66,76 | 46,37 | 97,52 |
| 15-HEPE | 130,30 | 58,87 | 116,95 |
| 12-HEPE | 70,93 ^a^ | 39,55 | 19,48 ^a^ |
| 12-HETE | 2386,88 | 1964,25 | 1576,00 |
| 9-HODE | 22627,87 | 27208,85 | 55478,78 |
| 13-HODE | 1167,91 | 898,24 | 2884,41 |
| 15-HETE | 2006,41 | 1414,53 | 4284,49 |
| 13-KODE | 740,61 | 862,38 | 1000,14 |
| 15-KETE | 223,51 | 225,34 | 266,36 |
| 11-HETE | 228,03 | 280,91 | 192,00 |
| 8-HETE | 0,00 | 0,00 | 0,00 |
| 17-HDHA | 347,82 | 170,40 | 420,26 |
| 4-HDHA | 0,00 | 0,00 | 0,00 |
| 12-KETE | 130,44 ^b^ | 198,57 ^b / cc^ | 68,32 ^cc^ |
| 5-HETE | 276,62 | 329,73 | 79,79 |
| 17-HDPA | 2093,65 | 1381,09 | 1913,79 |
| 17-oxo-DHA | 305,66 | 315,89 | 367,03 |
| 15-HETrE | 130,93 | 125,54 | 291,08 |
| 12-HETrE | 344,36 | 359,38 | 295,68 |
| 5-KETE | 2,06 | 4,76 | 0,00 |
| RVE1 | 0,00 | 0,00 | 0,00 |
| RVD3 | 0,00 | 0,00 | 0,00 |
| RVD2 | 0,00 | 0,00 | 0,00 |
| RVD1 | 0,00 | 0,00 | 0,00 |
| RVD4 | 0,00 | 0,00 | 0,00 |
| RVE4 | 4,31 ^a^ | 0,78 | 0,11 ^a^ |
| Maresin 1 | 0,00 | 0,00 | 0,00 |
| 10,17-DiHDHA (PDX) | 2,88 | 3,08 | 1,20 |
| RVD5 | 8,40 | 3,86 | 4,34 |
| Maresin 2 | 0,00 | 0,00 | 0,00 |
| 20-COOH-LTB4 | 0,00 | 0,00 | 0,00 |
| 20-OH-LTB4 | 0,00 | 0,00 | 0,00 |
| EXC4-EA | 0,00 | 0,00 | 0,00 |
| EXC4-G | 0,00 | 0,00 | 0,00 |
| EXC4 | 0,00 | 0,00 | 0,00 |
| EXD4 | 0,00 | 0,00 | 0,00 |
| LTC4 | 0,00 | 0,00 | 0,00 |
| LTD4 | 0,00 | 0,00 | 0,00 |
| LTE4 | 0,00 | 0,00 | 0,00 |
| LTB5 | 0,19 | 0,35 | 0,05 |
| 5(S),12(S)-DiHETE | 21,20 ^a^ | 19,94 ^c^ | 10,54 ^a / c^ |
| LTB4 | 0,00 | 0,00 | 0,00 |
| 12-oxo LTB4 | 0,03 | 0,00 | 0,79 |
| LTB3 | 0,00 | 0,00 | 0,00 |
| DGLA | 696,47 | 1779,40 | 1036,30 |
| PGB2 | 2,39 ^a / b^ | 0,41 ^b^ | 0,76 ^a^ |

Significance: ^a / b / c^ *p*-value < 0.05, ^aa / bb / cc^ *p*-value < 0.01 and ^aaa / bbb / ccc^ *p*-value < 0.001. Abbreviations: a, significance between CTL and LA10/75; b, significance between CTL and LA10/10; c, significance between LA10/10 and LA10/75; CTL, control; LA, linoleic acid.

**
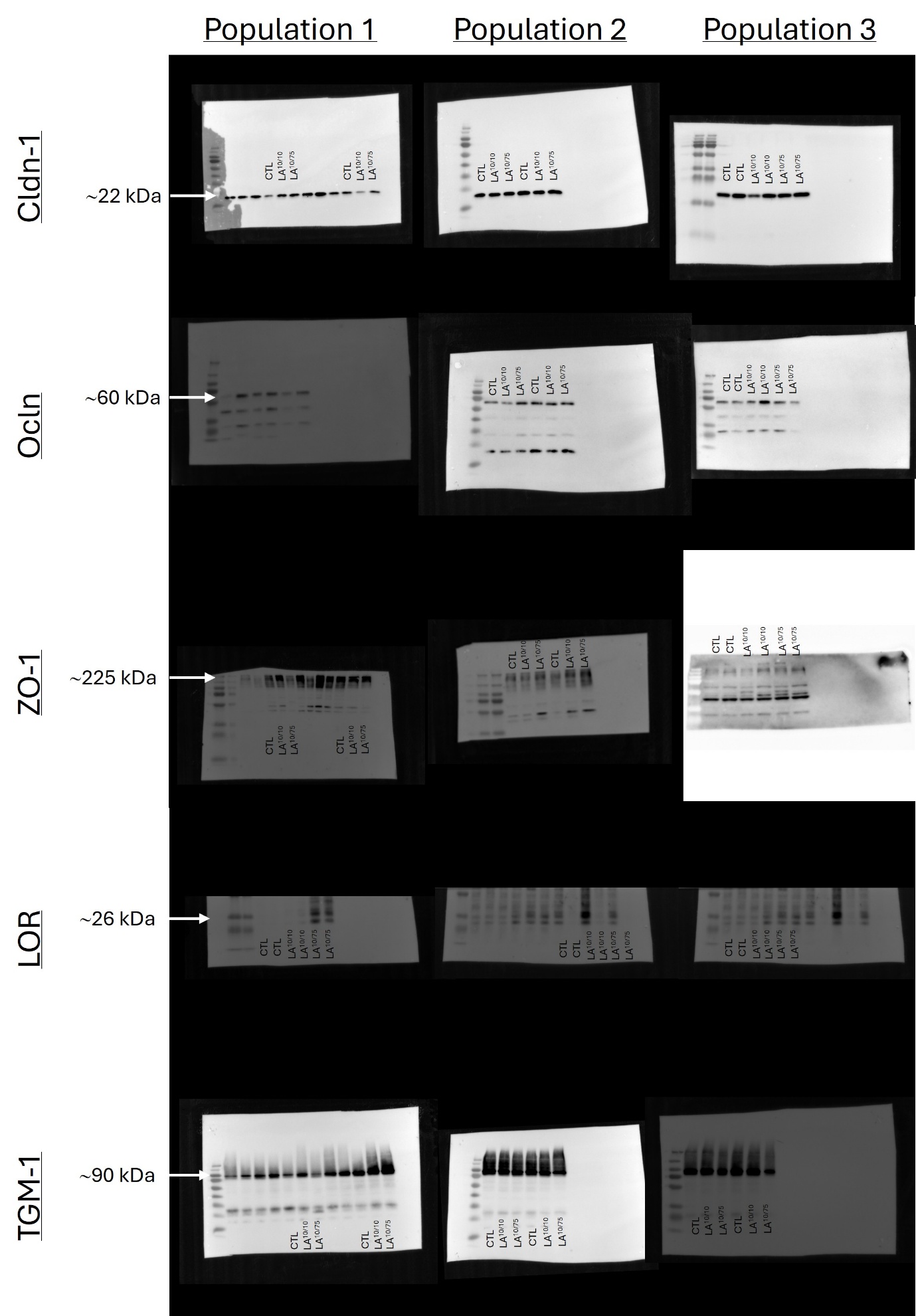
**

**Figure S5. Uncropped western blot membranes for all tested tight junction proteins and differentiation markers.**

**Table S2: Antibodies used for western blotting.**

| **Primary antibodies** | **Dilution** | **Incubation time** | **Source** | **Supplier** |
| --- | --- | --- | --- | --- |
| **CLDN-1** | 1:2000 | O/N | Rabbit IgG | ab15098, Abcam, Cambridge, MA, USA |
| **ZO-1** | 1:50 | O/N |  | 40-2200, Thermo Fisher Scientific, Eugene, OR, USA |
| **OCLN** | 1:1000 | O/N |  | ab216327, Abcam, Cambridge, MA, USA |
| **MAGL** | 1:2000 | O/N |  | ab124796, Abcam, Cambridge, MA, USA |
| **TGM-1** | 1:2000 | O/N |  | 12912-3-AP, Proteintech, Rosemont, IL, USA |
| **LOR** | 1:2000 | 1 hour |  | ab176322, Abcam, Cambridge, MA, USA |
| **Actin-β** | 1:30000 | 1 hour | Mouse IgG1 | A5441, Sigma, Oakville, ON, Canada |

**Table S3: Sequences of each primer used for RT-qPCR analyses.**

| **Gene** | **Primer** | **Sequence** | **Temperature (°C)** |
| --- | --- | --- | --- |
| **ABHD5** | Forward | 5’-CAG CAT CCA GTC CTT ACG ACC A-3’ | 57 |
|  | Reverse | 5’-GTT CAG TCC ACA GTG TCG CAG A-3’ |  |
| **ACTINβ** | Forward | 5’-CACCCTGAAGTACCCCATCG-3’ | 54 |
|  | Reverse | 5’-TGCCAGATTTTCTCCATGTCG-3’ |  |
| **ALOX12B** | Forward | 5’-TGC TGG AGA CAC ACC TCA TTG C-3’ | 58 |
|  | Reverse | 5’-GCC AAT GCT GTT GAT CTG GAC G -3’ |  |
| **ALOXE3** | Forward | 5’-GCT GCT CTT CAA TGC CAT CCC T-3’ | 58 |
|  | Reverse | 5’-TGT CGT GAA GGT CTT ATG GCA CC-3’ |  |
| **DAGLβ** | Forward | 5’-CAG CTC AAC TGT CAC TTC GGC T-3’ | 59 |
|  | Reverse | 5’-CAG AGC CAC TAA AAA CGG CAG C-3’ |  |
| **GADPH** | Forward | 5’-TGC ACC ACC AAC TGC TTA GC-3’ | 57 |
|  | Reverse | 5’-GGC ATG GAC TGT GGT CAT GAG-3’ |  |
| **PNPLA1** | Forward | 5’-CAG TCT GGA AGG AGC CAC ACA A-3’ | 58 |
|  | Reverse | 5’-GCT GAA ACA GGT GAC TCG CAT G-3’ |  |
| **SDR9C7** | Forward | 5’-CTA CTG CGT CTC CAA GTT TGG C-3’ | 57 |
|  | Reverse | 5’-GAG AAT GGC TGT CCG ATA GTT CC-3’ |  |
